# Exploring the link between depression, anxiety, and oral health-related quality of life: A cross-sectional study in 2 Arab countries

**DOI:** 10.1097/MD.0000000000046968

**Published:** 2026-01-02

**Authors:** Mohammed I. Alsaif, Abdullah S. Bin Rahmah, Abdallah Y. Naser

**Affiliations:** a Department of Periodontics and Community Dentistry, College of Dentistry, King Saud University, Riyadh, Saudi Arabia; b Department of Applied Pharmaceutical Sciences and Clinical Pharmacy, Faculty of Pharmacy, Isra University, Amman, Jordan.

**Keywords:** anxiety, depression, jordan, oral, quality of life, Saudi Arabia

## Abstract

Mental disorders such as depression affects the daily activity and performance. including self-care behaviors and personal hygiene. Oral health-related quality of life (OHRQoL) is a complex concept that includes social, psychological, and physical aspects. This study seeks to investigate the correlation between OHRQoL and depression and anxiety. This is an online cross-sectional survey study that was performed in Jordan and Saudi Arabia between September and November 2024. This research utilized 3 previously validated tools to achieve the study aim of examining the link between OHRQoL and depression and anxiety. The Oral Health Impact Profile (OHIP)-14, Patient Health Questionnaire-9, and General Anxiety Disorder-7. This study involved 2704 participants. Among our participants, the prevalence of moderate to severe depression was 56.0%, the prevalence of severe anxiety was 21.4%, and the mean OHIP score was 14.9 (standard deviation SD]: 12.6) out of 56, which implies a low to moderate impact on participants’ OHRQoL. Physical pain dimension showed the higher mean score with 2.8 (SD: 2.3); which reflects discomfort and pain caused by dental conditions. Functional limitation dimension showed the lowest mean score with 1.5 (SD: 1.9); which reflects practical difficulties caused by oral health issues (such as speech or taste impairment). Pearson correlation coefficient indicated a moderately positive correlation between OHIP score and Patient Health Questionnaire-9 score *R* = =0.395; 95% confidence interval: 0.363–0.426; *P*-value <.001) and a moderately positive correlation between OHIP score and General Anxiety Disorder-7 score *R* = =0.416; 95% confidence interval: 0.385–0.447; *P*-value <.001). Oral health issues demonstrated low to moderate impact on participants’ QoL. Physical pain was more noticeable issue that affected participants’ QoL due to their oral health issues compared to functional limitation dimension. Oral-related QoL is negatively affecting the psychological status of individuals due to its impact on their emotional well-being. Healthcare professionals are advised to give attention to both oral and mental health of their patients.

## 1. Introduction

Mental disorders such as anxiety and depression were increased in the last decades, and considered as the most common mental disorders in the United States.^[1]^ Moreover, the mental health disorders incidence and prevalence was increased significantly by COVID-19 pandemic specially in adults.^[2]^ Several factors associated with anxiety and depression in adulthood, for instance, lifestyle factors, socioeconomic factors, and other health-related factors.^[3,4]^

Mental disorders, including depression, affect daily activity and performance, such as personal hygiene and self-care behaviors. Anxiety can cause apprehension that influences day-to-day habits, resulting in the neglect of oral hygiene, and consequently, poor oral health-related quality of life (OHRQoL).^[5–7]^ In previous literature, abundant studies demonstrated that there is a strong association between OHRQoL and depression and anxiety.^[3,8–12]^ Specifically, research in South Korea reported a significant association between depressive episodes and poor elderly oral health.^[13]^ Another study conducted in Korea demonstrated that depression and oral dryness negatively affect OHRQoL.^[14]^

Together with oral impact on daily performance, OHRQoL is a complex concept that includes social, psychological, and physical aspects. This composite measure outlines people’s opinions about how dental health affects their general well-being and ability to carry out everyday tasks. Its assessment typically entails the utilization of multifaceted questionnaires.^[15]^ As a result, OHRQoL is a crucial tool for assessing how oral health issues influence quality of life (QoL) for people, emphasizing the necessity of improving oral health practices and management to maximize general well-being.^[16]^ Previous studies in the Middle east examined mental health among the general public before, during, and after COVID-19 pandemic.^[17–20]^ However, there are limited evidence on the association between mental conditions and OHRQoL. Therefore, this study seeks to investigate the correlation between OHRQoL and depression and anxiety.

## 2. Methods

### 2.1. Study design and population

This is an online cross-sectional survey study that was performed in Jordan and Saudi Arabia between September and November 2024. The study population for this research was individual from the general public of the 2 countries. The inclusion criteria were participants aged 18 years and over who presently reside in one of the 2 countries. This study did not exclude participants based on their gender, age, or socioeconomic status.

### 2.2. Sampling technique

In order to recruit the participants for this study from broad range of socioeconomic status within considerable time and efforts we implemented convenience sampling technique. This sampling technique recruits potential participants based on their availability and willingness to participate. The social media platforms (WhatsApp, Facebook, and Instagram) were used to invite the study participants to be involved in the study. The questionnaire was administered in Arabic language. The inclusion criteria were highlighted in the cover letter upon inviting them in order to facilitate participants who meet the inclusion criteria to participate only. The study participants were notified that there is no financial incentive based on their participation and that their information will be kept secured and anonymized.

### 2.3. Study tools

This research utilized 3 previously validated tools to achieve the study aim of examining the link between OHRQoL and depression and anxiety. The oral health impact profile (OHIP)-14, patient health questionnaire (PHQ)-9, and general anxiety disorder (GAD)-7 were used in this study.^[21–23]^ The PHQ-9 and GAD-7 scales utilize a 4-point Likert scale, ranging from “not at all” scored as zero to “nearly every day” scored as 4. The maximum score for the PHQ-9 scale is 27. A score ranging from 0 to 4 signifies “minimal depression,” 5 to 9 indicates “mild depression,” 10 to 14 reflects “moderate depression,” 15 to 19 denotes “moderately severe depression,” and 20 to 27 represents “severe depression.” The maximum score on the GAD-7 scale is 21. A score ranging from 5 to 9 signifies mild anxiety, 10 to 14 denotes moderate anxiety, and 15 to 21 reflects severe anxiety.^[20,22,23]^ The OHIP-14 questionnaire was originally developed by Slade GD. This questionnaire tool examines 7-dimensions related to the impact that oral health problems have on individuals’ QoL, which are functional limitation, physician; pain, psychological discomfort, physical disability, psychological disability, social disability, and handicap. The 14 questions are measured using 5-point Likert scale that range between never (scored as zero) and very often (scored 4). The maximum attainable OHIP-14 score is 56. The higher the score the higher the impact of oral health on individuals’ QoL (more serious oral health problems). The internal reliability coefficient (α) of OHIP-14 is 0.88 which is deemed good to excellent.^[21]^

### 2.4. Questionnaire piloting

Before the distribution of study questionnaire tools, they were examined by expert in oral health researchers to assess their suitability to achieve the study aim. Besides, they were asked about their clarity. They confirmed the items clarity and suitability for achieving the study aim. This was followed by pilot study on 40 participants from the general public who meet the study inclusion criteria to check the questionnaire understandability and ease of use.

### 2.5. Statistical analysis

The Statistical Package for Social Science Software (SPSS, Chicago) version 29 analyzed the data for this study. Continuous variables were presented using mean and standard deviation (SD). Categorical variables were presented as frequencies and percentages. The correlation between anxiety score, depression score, and OHIP-14 score was examined using Pearson correlation coefficient test. Multiple logistic regression analysis was conducted to identify predictors of anxiety and depression. The findings were presented as odds ratio with 95% confidence interval (CI). The significance level was assigned as *P*-value <.05.

## 3. Results

### 3.1. Participants’ demographic characteristics

A total of 2704 participants were involved in this study. Around half of them (53.9%) were females. The age group 31 to 40 years contributed for 28.6% of the study sample. The majority of the participants (81.3%) were single. Around 55.0% of the participants reported that they hold bachelor degree. Around one-third of the study participants (38.5%) reported that their family monthly income level is <700$. Around 41.1% of the participants reported that they are unemployed. Almost one-fifth of the study participants (18.5%) reported that they are smokers. Around one-tenth of the study participants (9.9%) reported that they have comorbidities history, Table 1.

**Table 1:** Participants’ demographic characteristics.

Variable	Overall		Jordan (n = 1684)		Saudi Arabia (n = 1020)	
	Frequency	Percentage	Frequency	Percentage	Frequency	Percentage
Gender						
Females	1458	53.9	762	45.2	696	68.2
Age						
18–23 yr	587	21.7	278	16.5	309	30.3
24–30 yr	678	25.1	467	27.7	211	20.7
31–40 yr	772	28.6	355	21.1	417	40.9
41–50 yr	278	10.3	117	6.9	161	15.8
51–60 yr	219	8.1	150	8.9	69	6.8
61 yr and older	170	6.3	89	5.3	81	7.9
Marital status						
Single	2197	81.3	1554	92.3	643	63.0
Married	440	16.3	120	7.1	320	31.4
Divorced	51	1.9	9	0.5	42	4.1
Widowed	16	0.6	1	0.1	15	1.5
Education level						
Diploma or lower	1086	40.2	720	42.8	366	35.9
Bachelor degree	1487	55.0	897	53.3	590	57.8
Higher education	131	4.8	67	4.0	64	6.3
Family monthly income level						
<700 $	1040	38.5	895	53.1	145	14.2
700–1500 $	680	25.1	522	31.0	158	15.5
1500–2000 $	408	15.1	146	8.7	262	25.7
More than 2000 $	576	21.3	121	7.2	455	44.6
Employment status						
Retired	73	2.7	24	1.4	49	4.8
Unemployed	1112	41.1	744	44.2	368	36.1
Working in healthcare sector	166	6.1	29	1.7	137	13.4
University student	970	35.9	773	45.9	197	19.3
Working in other sectors	383	14.2	114	6.8	269	26.4
Smoking status						
Smoker	500	18.5	318	18.9	182	17.8
Comorbidities history						
Yes	268	9.9	156	9.3	112	11.0

### 3.2. Depression, anxiety, and oral health-related quality of life profile

#### 3.2.1. Depression and anxiety

The prevalence of moderate to severe depression among our study sample was 56.0%. The prevalence of severe anxiety among study sample was 21.4%. The depression and anxiety status of the study sample is presented below in Table 2.

**Table 2:** Anxiety and depression status of the study sample.

Variable	Frequency	Percentage
PHQ-9 diagnosis based on score distribution		
Minimal or none	511	18.9
Mild depression	677	25.0
Moderate depression	719	26.6
Moderately severe depression	496	18.3
Severe depression	301	11.1
GAD-7 diagnosis based on score distribution		
Minimal anxiety	713	26.4
Mild anxiety	757	28.0
Moderate anxiety	654	24.2
Severe anxiety	580	21.4

#### 3.2.2. Oral health-related quality of life

The mean OHIP score for our study sample was 14.9 (SD: 12.6) out of 56; which indicates a low to moderate impact on participants’ QoL due to oral health issues. Physical pain dimension showed the higher mean score with 2.8 (SD: 2.3); which reflects discomfort and pain caused by dental conditions. Functional limitation dimension showed the lowest mean score with 1.5 (SD: 1.9); which reflects practical difficulties caused by oral health issues (such as speech or taste impairment). Table 3 presents OHRQoL score stratified by dimension.

**Table 3:** Oral health-related quality of life score stratified by dimension.

Variable	Mean score	Standard deviation
OHIP- 14 dimensions		
Functional limitation	1.5	1.9
Physical pain	2.8	2.3
Psychological pain	2.7	2.4
Physical disability	1.9	2.1
Psychological disability	2.4	2.3
Social disability	1.7	2.1
Handicap	1.8	2.1
Overall score	14.9	12.6

#### 3.2.3. Correlation between depression, anxiety, and oral health-related quality of life

Pearson correlation coefficient showed a moderately positive correlation between OHIP score and PHQ-9 score (*R* = 0.395; 95% CI: 0.363–0.426; *P*-value <.001). Pearson correlation coefficient showed a moderately positive correlation between OHIP score and GAD-7 score (*R*= 0.416; 95% CI: 0.385–0.447; *P*-value <.001).

#### 3.2.4. Predictors of severe anxiety and depression

Using multiple logistic regression analysis that adjusted for age, gender, marital status, income level, employment status, smoking status, and having comorbidities history, we identified that participants who have bachelor degree were less likely to have severe anxiety compared to others (AOR: 0.73; [95% CI: 0.58–0.92]) (*P*-value = .007). Besides, higher income level was associated with lower likelihood of experiencing severe anxiety 700–1500 $ (AOR: 0.74; [95% CI: 0.58–0.93]) (*P*-value = .010), 1500–2000 $ (AOR: 0.56; [95% CI: 0.41–0.76]) (*P*-value <.001), and more than 2000 $ (AOR: 0.49; [95% CI: 0.36–0.67]) (*P*-value <.001). Unemployed participants (AOR: 2.85; [95% CI: 1.36–5.95]) (*P*-value = .005) and those working in healthcare sector (AOR: 2.30; [95% CI: 1.08–4.89]) (*P*-value = .031) were more likely to have severe anxiety compared to others. Moreover, smokers (AOR: 1.47; [95% CI: 1.15–1.89]) (*P*-value = .002) and those who have comorbidity (AOR: 1.67; [95% CI: 1.24–2.26]) (*P*-value <.001) showed higher likelihood of having severe anxiety compared to others.

Concerning severe depression predictors, participants aged 24 to 30 years (AOR: 0.68; [95% CI: 0.50–0.91]) (*P*-value = .010) and 31 to 40 years (AOR: 0.47; [95% CI: 0.26–0.86]) (*P*-value = .014) were less likely to have severe depression compared to others. Besides, married participants were less likely (AOR: 0.62; [95% CI: 0.44–0.87]) (*P*-value = .005) to have severe depression compared to others. Similarly, higher income level was associated with lower likelihood of experiencing severe depression 700–1500 $ (AOR: 0.65; [95% CI: 0.53–0.80]) (*P*-value <.001), 1500–2000 $ (AOR: 0.45; [95% CI: 0.34–0.60]) (*P*-value <.001), and more than 2000 $ (AOR: 0.44; [95% CI: 0.33–0.58]) (*P*-value <.001). Unemployed participants (AOR: 3.38; [95% CI: 1.73–6.63]) (*P*-value <.001) and those working in healthcare sector (AOR: 2.55; [95% CI: 1.28–5.07]) (*P*-value = .008) were more likely to have severe depression compared to others. Moreover, smokers (AOR: 1.66; [95% CI: 1.32–2.09]) (*P*-value <.001) and those who have comorbidity (AOR: 2.05; [95% CI: 1.54–2.72]) (*P*-value <.001) showed higher likelihood of having severe depression compared to others.

## 4. Discussion

The primary aim of this study was to assess the OHIP score among Jordanians and Saudis and to examine the association between OHIP and mental health outcomes, specifically depression and anxiety. Our findings demonstrated a significant association between OHIP score and both depression and anxiety, indicating that poorer oral health is linked to higher anxiety and depression levels.

The prevalence of moderate to severe depression (total score ≥ 10) in our study sample was 56.0%, while the prevalence of severe anxiety (total score ≥ 15) among study sample was 21.4%. In contrast, another study assessing depression and anxiety levels reported a prevalence of 10.9% for moderate to severe depression and 15.5% for anxiety.^[24]^ This discrepancy may be attributed to differences in the characteristics of study populations. Several studies have found that a variety of comorbidities are related with depression and anxiety. For example, systemic illness includes obesity and difficulty sleeping. Depression has a significant impact on dental health due to a range of behavioral and biological factors, including the adoption of risky habits such as frequent alcohol intake, smoking, excessive fat and sugar intake, and sedentary lifestyles.^[25–27]^

The mean OHIP score for our study sample was 14.9 (SD: 12.6) out of 56; which implies a low to moderate impact on participants’ QoL due to oral health issues. Physical pain dimension showed the higher mean score with 2.8 (SD: 2.3); which reflects discomfort and pain caused by dental conditions. Functional limitation dimension showed the lowest mean score with 1.5 (SD: 1.9); which reflects practical difficulties caused by oral health issues (such as speech or taste impairment). Similarly, A study conducted in Poland among 150 subjects reported a mean OHIP-14 score of 8.72 (13.39), with physical pain also being the highest-scoring domain (1.81, SD:2.12). However, in contrast to our findings, the lowest score in the Polish study was for social disability domain (0.93, SD: 1.94).^[28]^ In addition to the population characteristics, the differences in the findings could also be attributed to the variation in the cultural perception of the oral health. Clinically, the greater physical pain levels seen in both our and the Polish studies demonstrate the major impact of oral discomfort on QoL. Oral health disorders, particularly those that cause discomfort, can have an impact on daily functioning and psychological well-being, leading to avoidance of necessary dental care and additional consequences. The lower functional limitation scores indicate that, while discomfort is noticeable, direct interference with tasks such as talking and eating may be less severe. These findings highlight the significance of pain management and preventive techniques in dental treatment for improving patient QoL, particularly in populations with limited access to routine dental services.^[29]^

The Pearson correlation coefficient indicated a moderately positive correlation between OHIP score and PHQ-9 score (*R* = 0.395; 95% CI: 0.363–0.426; *P*-value <.001). This reflects that individuals who have poorer OHRQoL tend to have higher depression level. Similarly, a study conducted in Germany that assessed the association between OHIP and depression using the same scale found a moderately positive correlation (*R* = 0.33).^[3]^ Another study also demonstrated a similar association between depression and poor OHRQoL.^[30]^ These similarities across studies from different countries suggest that overall oral health significantly impacts life satisfaction and QoL, which, in turn, contributes to mental health. including depression.^[31]^ Clinically, oral health issues, particularly those that cause pain, can result in low QoL, anxiety, and depression. Chronic inflammation induced by oral diseases, like as periodontitis, can also cause changes in hormone and neurotransmitter levels in the brain, resulting in depression.^[27,32,33]^

Pearson correlation coefficient showed a moderately positive correlation between OHIP score and GAD-7 score (*R* = 0.416; 95% CI: 0.385–0.447; *P*-value <.001). This reflects that individuals who have poorer OHRQoL tend to have higher anxiety level. In contrast to our findings, a study in Germany reported a low overall association between OHIP and GAD-7 score, with a moderate positive association observed specifically in males.^[3]^ This discrepancy may be attributed to the difference in the population characteristics, particularly the stronger association found in males compared to females. Several studies demonstrated that poor teeth care and embarrassed due to teeth, lower the self-esteem, which, in turn increases the anxiety level.^[34–36]^ Clinically, the significant correlation between poor OHRQoL and anxiety levels indicates the odds of mental health such as anxiety and depression are higher in patients experiencing pain with oral health or dental procedure. Furthermore, studies found that dental anxiety is a psychological disorder that may affects patients’ physical health.^[37,38]^

In our study, the likelihood of experiencing severe anxiety for participants with a bachelor’s degree was lower than for others. Besides, higher income level was associated with lower likelihood of experiencing severe anxiety and depression. Unemployed participants and those working in healthcare sector were more likely to have severe anxiety and depression. Moreover, smokers and those who have comorbidity showed higher likelihood of having severe anxiety and depression. Besides, participants aged 24 to 30 years and 31 to 40 years were less likely to have severe depression. Besides, married participants were less likely to have severe depression. A recent study by Kim C. identified socioeconomic aspects affecting depression using machine learning and found that depression scores were significantly predicted by weekly working hours, marital status, age, and monthly income. Kim C.’s study unexpectedly showed that higher income levels were associated with higher depression scores, which might be related to raised stress. Kim C.’s study emphasizes that the correlation between socioeconomic status and depression is complex. Moreover, they highlight the requirement for mental health policies to address psychological and economic stressors, primarily for individuals at high-risk.^[39]^

Previous literature showed that higher levels of education and income are less likely to experience severe anxiety and depression. This could be justified due to the fact that individuals with higher levels of education and income have superior coping resources and social advantages.^[40,41]^ In contrast, unemployment and employment in high-stress environments, such as healthcare are associated with higher anxiety and depression levels.^[42]^ Moreover, being smoker and having chronic conditions history showed higher probability of developing severe anxiety and depression.^[43]^ Our findings highlighted a significant correlation between OHRQoL and mental health, such as depression and anxiety. Clinically, this finding supports the importance of patient care, where dentists and dental care specialist should consider the psychological well-being of their patients, especially those with poor oral health. Screening for mental health issues like depression and anxiety during dental visits could enable early intervention and improve both oral health outcomes and overall QoL.

There were several potential limitations to this study. First, a cross-sectional study design such as self-reported questionnaire, may introduce bias, since the participants may over or underestimate their oral and mental health status. Second, this study is prone to selection bias as convenience sampling via social media may overrepresent online-active individuals while under-representing older or lower-income groups. Therefore, the majority of our sample were aged <40 years old, this may affect the accuracy and generalizability of our findings limiting applicability to older individuals with greater oral health issues. Besides, measurement bias due to self-reported data may lead to over or underestimation due to recall and social desirability bias. Lastly, no dental examinations were conducted in this study, relying entirely on self-reported symptoms, which lack the clinical validation.

Future longitudinal research should focus on the relationship between oral and mental health, determining whether poor oral health leads to depression and anxiety or vice versa. Future studies should include clinical validation for the patients and confirming their oral health profile through healthcare professionals. Future studies should implement better sampling technique to enhance the generalizability of the study findings. Additionally, interventions studies should also be considered to assess oral health promotion programs effects on mental health.

## 5. Conclusion

Oral health issues demonstrated low to moderate impact on participants’ QoL. Physical pain was more noticeable issue that affected participants’ QoL due to their oral health issues compared to functional limitation dimension. This highlights the importance of giving more attention to pain relief to improve individuals’ oral-related QoL. Furthermore, the observed moderately positive correlation between anxiety score, depression score, and OHIP score highlights the psychological impact of oral health on individuals. Moreover, this demonstrates that oral-related QoL is negatively affecting the psychological status of individuals due to its impact on their emotional well-being. Healthcare professionals are advised to give attention to both oral and mental health of their patients.

## Author contributions

**Conceptualization:** Mohammed I. Alsaif, Abdullah S. Bin Rahmah.

**Data curation:** Mohammed I. Alsaif, Abdullah S. Bin Rahmah, Abdallah Y Naser.

**Formal analysis:** Mohammed I. Alsaif, Abdallah Y Naser.

**Funding acquisition:** Mohammed I. Alsaif.

**Investigation:** Mohammed I. Alsaif, Abdullah S. Bin Rahmah, Abdallah Y Naser.

**Methodology:** Mohammed I. Alsaif, Abdallah Y Naser.

**Project administration:** Mohammed I. Alsaif.

**Resources:** Mohammed I. Alsaif, Abdullah S. Bin Rahmah, Abdallah Y Naser.

**Software:** Mohammed I. Alsaif, Abdallah Y Naser.

**Supervision:** Mohammed I. Alsaif.

**Validation:** Mohammed I. Alsaif, Abdullah S. Bin Rahmah, Abdallah Y Naser.

**Visualization:** Mohammed I. Alsaif, Abdullah S. Bin Rahmah, Abdallah Y Naser.

**Writing – original draft:** Mohammed I. Alsaif, Abdullah S. Bin Rahmah, Abdallah Y Naser.

**Writing – review & editing:** Mohammed I. Alsaif, Abdullah S. Bin Rahmah, Abdallah Y Naser.
